# Comparing GlideScope Video Laryngoscope and Macintosh Laryngoscope Regarding Hemodynamic Responses During Orotracheal Intubation: A Randomized Controlled Trial

**DOI:** 10.5812/ircmj.12334

**Published:** 2014-04-05

**Authors:** Ali Reza Pournajafian, Mohammad Reza Ghodraty, Seyed Hamid Reza Faiz, Poupak Rahimzadeh, Hamidreza Goodarzynejad, Enseyeh Dogmehchi

**Affiliations:** 1 Department of Anaesthesiology, Firoozgar Hospital, Iran University of Medical Sciences, Tehran, IR Iran; 2 Department of Anaesthesiology, Rasoul-Akram Medical Center, Iran University of Medical Sciences, Tehran, IR Iran; 3 Department of Research, Tehran Heart Center, Tehran University of Medical Sciences, Tehran, IR Iran

**Keywords:** Laryngoscopes, Intubation, Hemodynamic Responses

## Abstract

**Background:**

To determine if the GlideScope® videolaryngoscope (GVL) could attenuate the hemodynamic responses to orotracheal intubation compared with conventional Macintosh laryngoscope.

**Objectives:**

The aim of this relatively large randomized trial was to compare the hemodynamic stress responses during laryngoscopy and tracheal intubation using GVL versus MCL amongst healthy adult individuals receiving general anesthesia for elective surgeries.

**Patients and Methods:**

Ninety five healthy adult patients with American Society of Anesthesiologists physical status class I or II that were scheduled for elective surgery under general anesthesia were randomly allocated to either Macintosh or GlideScope arms. All patients received a standardized protocol of general anesthesia. Hemodynamic changes associated with intubation were recorded before and at 1, 3 and 5 minutes after the intubation. The time taken to perform endotracheal intubation was also noted in both groups.

**Results:**

Immediately before laryngoscopy (pre-laryngoscopy), the values of all hemodynamic variables did not differ significantly between the two groups (All P values > 0.05). Blood pressures and HR values changed significantly over time within the groups. Time to intubation was significantly longer in the GlideScope (15.9 ± 6.7 seconds) than in the Macintosh group (7.8 ± 3.7 sec) (P< 0.001). However, there were no significant differences between the two groups in hemodynamic responses at all time points.

**Conclusions:**

The longer intubation time using GVL suggests that the benefit of GVL could become apparent if the time taken for orotracheal intubation could be decreased in GlideScope group.

## 1. Background

Laryngoscopy and passage of endotracheal tube through the larynx can lead to sympathetic stimulation and adverse effects in the physiological systems (1-3). In particular the adverse effects on the cardiovascular system commonly manifested as hypertension, tachycardia or arrhythmia (4, 5). These hemodynamic responses, that have been widely documented in many studies in various patient groups (4), are mostly short-lived and well tolerated by healthy individuals. However, they can be dangerous in susceptible patients resulting in morbidity and mortality (6), and can be detrimental in patients with cardiovascular disease (4, 7, 8). The magnitude of the hemodynamic responses during laryngoscopy and tracheal intubation is correlated with the degree of manipulation in oropharyngolaryngeal structures (9).

**Figure 1.**
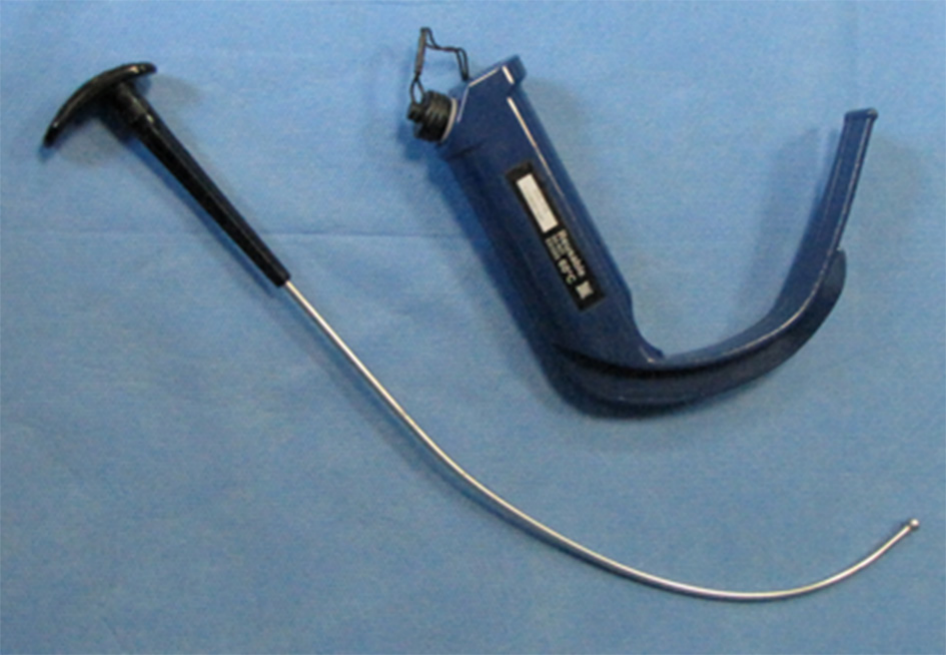
A GlideScope Video Laryngoscope With Its Specific Rigid Stylet

The Macintosh laryngoscope (MCL) has been the ‘gold standard’ device for direct laryngoscopy and tracheal intubation since its invention by Foregger in the 1940s (10). A relatively high forward and upward force is applied on the MCL handle to visualize glottis through aligning oral, pharyngeal and laryngeal axes. Tracheal intubation performed by intubating devices using indirect (video) laryngoscopy need comparatively less degree of manipulation of the airway (11); thus at least in theory, we expect less hemodynamic stress response by using such devices. The GlideScope ® video laryngoscope (GVL; Verathon Medical, Bothell, USA) is a reusable biomedical device consisting of a blade with a 60° curvature and a handle in one piece which is similar in technique to conventional laryngoscopy (Figure 1). However, it does not require alignment of the oropharyngeal axis to visualize the vocal cords. According to the manufacturer's data, due to its design, less upward lifting force during laryngoscopy and intubation is required (approximately 0.5 to 1.5 kg of force) than with direct laryngoscopy whereas the maximum force exerted by the laryngoscope blade on the base of the tongue is reported to be approximately 4 to 5 kilograms during conventional laryngoscopy (4). Previously, several investigators compared hemodynamic responses to tracheal intubation by GVL and MCL with conflicting results (12-16).

## 2. Objectives

The aim of this relatively large randomized trial was to compare the hemodynamic stress responses during laryngoscopy and tracheal intubation using GVL versus MCL amongst healthy adult individuals receiving general anesthesia for elective surgeries.

## 3. Patients and Methods

**Figure 2.**
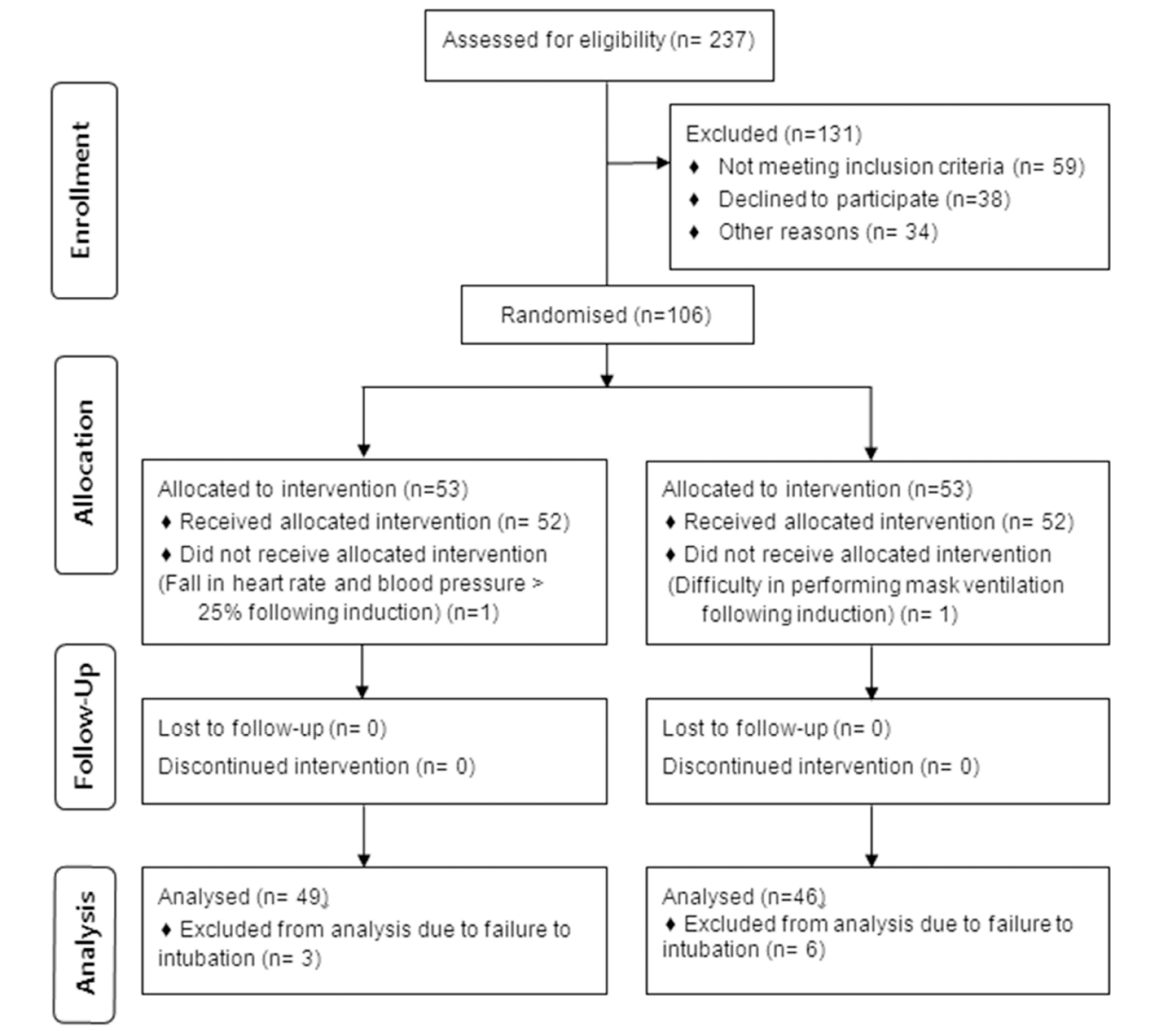
Flow Diagram of Patients Recruitment

This randomized clinical trial was conducted with approval of the Iran University of Medical Sciences (IUMS) Institutional Review Board (IRB) (on 11 January 2012), and registered with the www.irct.ir protocol registration system (IRCT201111264969N4). Between February and September 2012, this study was conducted at Firoozgar Teaching Hospital affiliated to IUMS, Tehran, Iran, and all the procedures are in accordance with the Declaration of Helsinki. After obtaining written informed consent from the patients, 106 consecutive patients were enrolled in this randomized clinical trial study (Figure 2). All the patients were scheduled for elective surgery under general anesthesia with American Society of Anesthesiologists physical status class (ASA) I or II who aged between 18 and 60. Patients with hypertension (history of hypertension or blood pressure (BP) > 140/90 mmHg on examination), lung disease, cardiovascular disease, cervical spine disease, gastro-esophageal reflux disease, and those in whom difficulty in intubation could be anticipated, such as patients with history of difficult intubation or laryngoscopy, those with BMI >30, short thyromental distance (< 6 cm), upper lip bite test (ULBT) grade III, and Mallampati score ≥ III were excluded from the study. Other exclusion criteria included history of any regular drug intake or allergy to any anesthetic medications, decrease in O_2_ saturation to ≤ 94% during ventilation or intubation, difficulty with mask ventilation during anesthesia, and intubation failure defined as patients requiring more than one attempt to achieve successful intubation, intubations that require more than 30seconds to be performed and also need for another person to complete the procedure.

All laryngoscopy and intubation procedures were performed by a single investigator (a fourth year anesthesiology resident with about 4 years’ experience in using Macintosh laryngoscopy who hereafter referred to as “the investigator”). Prior to starting the study, the investigator had no previous experience of intubation using video laryngoscope. The investigator performed 20 successful intubations with GVL under supervision of at least one of the two attending anesthesiologists at our hospital. After these first 20 cases that were not included in our study, the competency of investigator was approved to independently perform intubations using a video laryngoscope. The same investigator preoperatively recorded patients' characteristics and airway assessments. Modified Mallampati score was recorded while the patient sitting with mouth open and tongue protruded. Thyromental distance was measured as the distance between the anterior chin and the thyroid notch while the patient's neck was fully extended. All the patients were also asked to bite their upper lip and ULBT was performed (17). The allocation sequence was generated by a random allocation table in permuted blocks of four. A nurse not involved in the study or in the care of the patient assigned participants to one of the two study groups. The numbered opaque sealed envelopes that contained patient allocation were opened at the time of randomization. Patients were randomized into two groups: laryngoscopy and tracheal intubation performed with Macintosh blade (size 3 in women and size 4 in men) (Macintosh group) or with GVL (GlideScope group). When there was no response to single-twitch stimulation, the trachea was intubated with an endotracheal tube size 7 for women and 8 for men.

Patients entered the operating room after at least 8 hours overnight fasting, and were randomly allocated to either Macintosh or GlideScope arm. Five minutes before arrival in the operating room, all patients received an intravenous standardized premedication of fentanyl 3 mcg/kg, and midazolam 0.02 mg/kg. All the patients received 5 cc/ kg isotonic crystalloid solution and standard monitors were attached to them. Using an appropriate cuff size, all patients were investigated with the same calibrated and checked indirect arterial pressure machine. Intra-operative monitoring included pulse oximetry, electrocardiogram, and non-invasive arterial pressure. All the patients were preoxygenated with 100% oxygen through a face mask for three minutes, then general anesthesia was induced with intravenous administration of thiopental 5 mg/kg, and atracurium 0.6 mg/kg. Systolic BP, diastolic BP, mean arterial pressure (MAP), and heart rate (HR) were recorded at numerous intervals as follow: baseline, after induction of anesthesia, just before laryngoscopy, one, three, and five minutes after intubation and fixation of endotracheal tube. Pulse oximeters were used as heart rate monitors in our operating rooms. The rate-pressure product (RPP), product of HR and systolic BP, was calculated at all the measuring points. A second person (a single operating room technician) acted as the time keeper using a digital chronometer. Total intubation time (in seconds) was defined as the time from insertion of the assigned intubating device into the mouth up to the time the tracheal tube positioned between vocal cords. The polyvinyl chloride tracheal tubes with an internal diameter of 7.0 to 8.0 mm were used for all the patients. In the GlideScope group, an intubating stylet was adequately lubricated with a silicone-based fluid and inserted into the tracheal tube. The distal end of a styletted tracheal tube was lubricated and bent anteriorly to an angle of 60° which corresponded to the specially designed GlideScope blade with a 60° curvature.

### 3.1. Statistical Analysis

Statistical calculations were performed by Statistical Package for Social Sciences (SPSS) version 15.0 (SPSS Inc., Chicago, IL, USA) and GraphPad Prism® 5 (GraphPad Software, La Jolla, CA, USA). As a power analysis based on a previous study (12) suggested, a sample size of 46 patients for each group was required to achieve a power of 90% at a 0.05 level of significance for detection of 8 beats per minute or 10 mmHg differences in paired hemodynamic data. Hypothesizing that the possibility of intubation failure was approximately 10%, we recruited 106 patients in total to account for possible drop-outs. While the mean differences regarding the age, weight, height and BMI among two groups were compared by independent student’s t-test, the Mann-Whitney U-test was applied for the evaluation of non-normally distributed data (time required for total intubation time). The categorical variables in the two groups were analyzed using Chi-square test or Fisher's exact test as appropriate. For the analysis of hemodynamic responses to intubation including HR, RPP, and systolic, diastolic and mean blood pressures, a repeated-measures analysis of variance (ANOVA) was used.

This test included within-subjects factors (hemodynamic changes over time) and one between-subjects factor (intubation technique). The within-subjects factor of time interval had six levels: baseline; after induction of anesthesia; just before laryngoscopy; as well as one minute, three minutes, and five minutes after intubation. The between subjects factor of intubation technique had two levels: group Macintosh and group GVL. Initial exploratory analyses were conducted to ascertain if the data met the assumptions of ANOVA: normality; homogeneity of variance; and sphericity.

Normality of the distribution was examined in several ways-visually (in the form of box plots and histograms) and statistically using measures of central tendency. All methods revealed that the data deviated slightly from a normal distribution in two groups across all time intervals. However, these deviations were predominantly slight. Extensive deviations from normality were found only in the time required for total intubation time where it skewed in a positive direction. Homogeneity of variance was tested using Levene’s Test for equality of error variance. Mauchly’s sphericity test was used to test for the condition of sphericity. When the test indicated that the assumption of sphericity had been violated (P< 0.05), the degrees of freedom were corrected using Greenhouse-Geisser estimates of sphericity. The changes in hemodynamic parameters in “two consequent times” within group were analyzed using paired Student’s t test. All tests were two-sided and a P-value <0.05 was considered statistically significant.

## 4. Results

One patient in the GlideScope group encountered difficulty in performing mask ventilation following induction of anesthesia, and one patient in Macintosh group encountered fall in HR and BP more than 25% following induction of anesthesia. These two patients were withdrawn from the study and treated appropriately. A total of nine patients (8.5%) in two groups were excluded from statistical analysis of the data due to failure in intubation. The total incidence of intubation failure was not statistically different in the GlideScope group as compared to Macintosh group (11.5% vs. 5.8%, respectively; P= 0.488).

**Figure 3.**
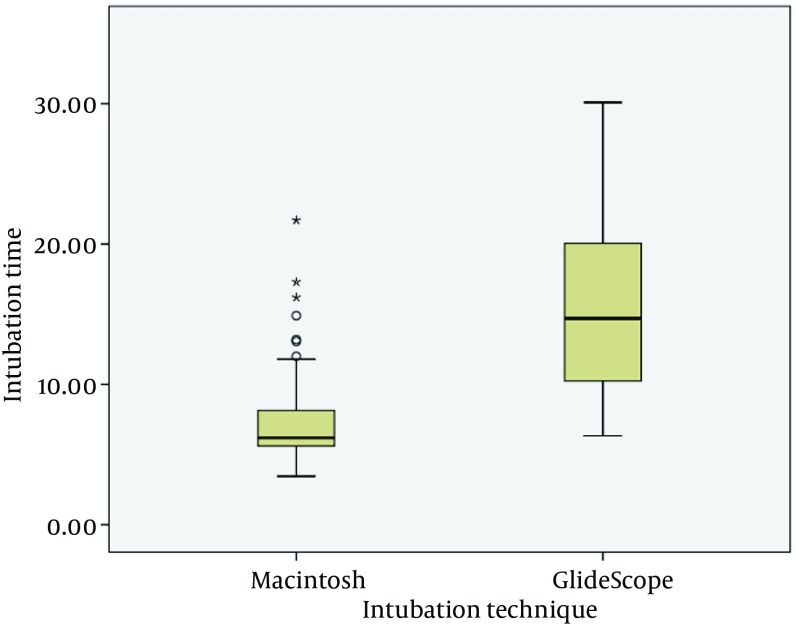
The Box-plot Graph of Comparison of Intubation Time (Seconds) Between Macintosh Group and Glidescope Group

A total of 95 patients were included in the statistical analysis of data. Overall, the patients’ mean age was 35 years and 38 (40%) were men. Patients ranged in body weight from 45 kg to 90 kg and height from 150 cm to 188 cm. The Macintosh and GlideScope groups were comparable with respect to baseline characteristic data except for that the patients in Macintosh group more likely had ASA I scores (Table 1). In all patients tracheal intubation was successful in the first attempt within 30 seconds. As seen in Figure 3, time to intubation was significantly longer in the GlideScope group (15.9 ± 6.7 seconds) than in the Macintosh group (7.8 ± 3.7 sec) (P < 0.001).

**Table 1.** Baseline Characteristics of the Study Population (n= 95) ^a^

	All	Macintosh (n = 49)	GlideScope (n = 46)	P Value ^b^
**Age, y**	34.8 ± 11.1	33.7 ± 10.6	36.1 ± 11.6	0.292
**Body mass index, kg/m** ^**2**^	24.5 ± 3.4	24.1 ± 3.3	24.9 ± 3.5	0.226
**Height, cm**	166.7 ± 8.2	165.9 ± 7.5	167.5 ± 8.9	0.345
**Weight, kg**	67.9 ± 9.8	66.2 ± 9.8	69.7 ± 9.1	0.079
**Sex**				0.503
Male	38 (40.0)	18 (36.7)	20 (43.5)	
Female	57 (60.0)	31 (63.3)	26 (56.5)	
**Mallampati class**				0.133
I	72 (75.8)	34 (69.4)	38 (82.6)	
II	23 (24.2)	15 (30.6)	8 (17.4)	
**ASA physical status **				0.008
I	80 (84.2)	46 (93.9)	34 (73.9)	
II	15 (15.8)	3 (6.1)	12 (26.1)	
**Upper lip bite test **				0.383
I	66 (69.5)	36 (73.5)	30 (65.2)	
II	29 (30.5)	13 (26.5)	16 (34.8)	

^a^ Data are presented in Mean ± SD or No. (%).

^b^ P values for Macintosh vs. GlideScope ASA (American Society of Anesthesiologists).

**Figure 4.**
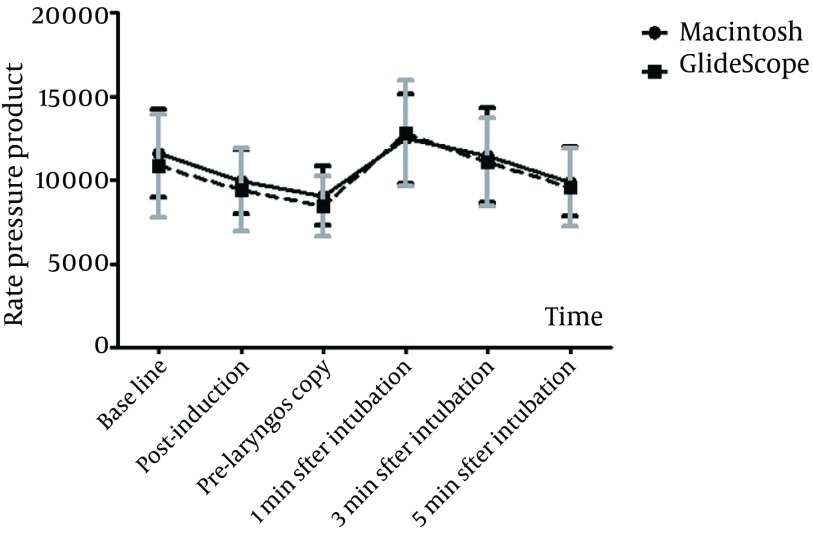
Changes of Mean Rate Pressure Product (Rpp) Before and After Intubation (Mean ± SD) in Two Various Laryngoscopy Methods

After induction of anesthesia, a significant reduction in systolic BP, diastolic BP, MAP, and RPP were recorded in both groups from their respective baseline values but HR remained unchanged (Table 2). Immediately before laryngoscopy (pre-laryngoscopy), the values of all hemodynamic variables did not differ significantly between the two groups (All p-values > 0.05). Blood pressures and HR values changed significantly over time within the groups. After intubation, arterial pressure increased significantly in both groups to a peak level observed at 1 minute post-intubation, at which point it was significantly greater than pre-laryngoscopy level in both groups (p < 0.05). However, there were no significant differences between the two groups in hemodynamic responses at all time points (Table 2 and Figure 4).

**Table 2.** Comparison of Hemodynamic Changes Associated With Orotracheal Intubation Between the Two Groups ^a, b^

Variables	Baseline	Post-induction	Pre-laryngoscopy	After Intubation, min			P Value
				1	3	5	
**Systolic BP, mmHg**							0.863
Macintosh	125.3 ± 10.8	111.5 ± 12.9 ^c^	109.1 ± 14.1	135.2 ± 19.5 ^d^	128.7 ± 16.8	118.3 ± 13.9	
GlideScope	127.0 ± 12.3	109.4 ± 16.7 ^c^	107.0 ± 14.5	140.6 ± 20.8 ^d^	128.5 ± 12.9	118.2 ± 16.6	
**Diastolic BP, mmHg**							0.410
Macintosh	80.2 ± 9.0	70.5 ± 12.1 ^c^	67.4 ± 12.6	91.0 ± 14.6 ^d^	86.8 ± 16.0	77.3 ± 12.3	
GlideScope	80.2 ± 7.4	66.5 ± 12.9 ^c^	66.0 ± 10.9	94.5 ± 13.0 ^d^	82.2 ± 13.0	72.7 ± 12.1	
**Mean AP, mmHg**							0.811
Macintosh	94.5 ± 8.7	83.8 ± 11.4 ^c^	80.2 ± 11.8	105.4 ± 16.3 ^d^	99.8 ± 15.2	90.5 ± 11.9	
GlideScope	94.4 ± 8.1	82.3 ± 14.8 ^c^	79.0 ± 11.3	110.4 ± 15.1 ^d^	97.4 ± 15.1	88.1 ± 12.0	
**Heart rate, beat/min**							0.160
Macintosh	92.0 ± 16.1	88.9 ± 13.5	83.1 ± 12.1	92.1 ± 14.3 ^d^	88.5 ± 14.9	83.4 ± 12.8	
GlideScope	84.5 ± 18.7	86.0 ± 17.2	79.0 ± 13.1	90.5 ± 15.0 ^d^	85.8 ± 15.0	80.6 ± 14.9	

^a^ Abbreviations: AP, arterial pressure; BP, blood pressure; SD, standard deviation.

^b^ Data are presented as Mean ± SD.

^c^ P value < 0.05 as compared to respective baseline values.

^d^ P value < 0.05 as compared to respective pre-laryngoscopy values.

## 5. Discussion

The hemodynamic changes as a result of laryngoscopy and intubation typically initiate within seconds of laryngoscopy (stimuli to oropharynx), and there are further hemodynamic responses with the passage of tracheal tube (stimuli to larynx and trachea) peaking in 1-2 minutes and lasting for 5 minutes (18). The GVL, a video laryngoscope developed to address difficult airways (18), is recommended by its manufacturer for intubation in all grades of difficulties. Thanks to its special blade, the GVL can reduce the mechanical stimulus to oropharyngolaryngeal structures during orotracheal intubation, and flatter and more uniform pressure distribution is produced upon the blade (19). Moreover, it requires less neck movement for intubation (20) thereby decreasing the potential for hemodynamic stimulation (21). Therefore, theoretically undesirable hemodynamic responses to intubation should be attenuated via using a GVL.

In 2007, Xue et al. (12) reported for the first time that there is no difference in hemodynamic responses to tracheal intubation between the GVL and MCL groups in 57 adults, ASA physical status I patients. The investigators postulated that the manipulation due to stylet, applied only in the GVL group, resulted in higher stimulus to the larynx and trachea, offsetting the decreased force required for laryngoscopy. In a later study, Siddiqui et al. (13) standardized the technique of intubation, using an intubating stylet in all the three groups of patients randomized to undergo intubation by GVL, Trachlight or direct laryngoscopy. They observed no difference in hemodynamic response between the groups and explained this by the fact that there is a growing body of evidence which indicates tracheal irritation rather than laryngeal irritation is the main stimulating factor for hemodynamic responses (18, 22-24). In our study, in agreement with the findings by the majority of previous studies (12-14, 25-27), there was no statistically significant difference between GlideScope group, in whom the GVL was used, and Macintosh group, in whom the direct laryngoscope was used, in BP and HR 1, 3, and 5 minutes after tracheal intubation. In contrast, several studies have demonstrated that tracheal intubation performed by devices using indirect laryngoscopy led to significantly lower hemodynamic responses (9, 28), and there is at least one report emphasizing the advantages of GVL over MCL to minimize hemodynamic responses in ASA class I and II patients (16). One possible reason for these conflicting results could be limited statistical power due to the modest sample size in most of these trials (n = 40) which may have played a role in limiting the significance of some of the statistical comparisons conducted. Moreover, confounders such as age, comorbidities, drug history, and type of induction medications could not definitively be excluded. Given the high statistical power of 90% for the present study, it is unlikely that the results to be affected by confounding factors or our negative findings to be attributed to a limited sample size.

In various studies, the GlideScope required a longer time for intubation than that of direct laryngoscopy with Macintosh (18, 29, 30); this increased duration can potentially be associated with more hemodynamic changes (18). Our results showed that total intubation time was significantly longer in GlideScope group than in Macintosh group but it did not result in greater hemodynamic response. A recent meta-analysis demonstrated that the time to intubation with GlideScope was shorter than with Macintosh when a novice or less experienced expert was performing the procedure; however, the author stated that these findings must be interpreted with caution because there were only two studies in this subgroup (31).The reason that intubation with the GVL led to similar hemodynamic changes to the laryngoscope, despite less lifting stimulation from the laryngoscope, could be, first, that the stimulation due to the passage of tracheal tube through the vocal cords has a greater impact on BP and HR than that due to the laryngoscope. In prior studies, there were greater hemodynamic responses when endotracheal intubation and laryngoscopy were performed in combination than when just lifting with the laryngoscope and this was attributed to the greater irritation on the respiratory tract from the tracheal tube than from the laryngoscope (18, 22-24). Second, it is possible that the longer intubation time in GlideScope group resulted in exaggerated cardiovascular responses to tracheal intubation (4, 32) which counterbalance the effect of reduced laryngoscope-lifting force. Several explanations for a longer time required for tracheal intubation with GVL compared with MCL have been discussed elsewhere (14, 33). Third, patients with anticipated difficult airway were excluded in this study. Hence, the differences in hemodynamic responses between the groups may be less noticeable. In patients with difficult airway more pressure from the laryngoscope is applied during tracheal intubation, and hemodynamic responses owing to the laryngoscope could be more intensive (34). Fourth, in this study only normotensive patients were included while investigators reported that there were no differences in BP in normal patients when applying lightwand and laryngoscope in tracheal intubation, but in hypertensive patients the application of the lightwand in intubation resulted in a lower rise in BP than that in the group in whom the laryngoscope was applied (9).

RPP is a sensitive index of myocardial oxygen consumption (mVO2) even in patients with hemodynamic changes under anesthesia, and surgery. A value of more than 22000 is generally associated with myocardial ischemia (12). In our study groups no patient had a RPP of more than 21000 except for one patient in GlideScope group who had a value of 22632 with no complications. This finding indicates that in anesthetized healthy adult patients the circulatory responses to orotracheal intubation using a GVL or a MDL is not accompanied by myocardial ischemia as a result of increased oxygen consumption.

There are several limitations to our study. First, since it was impossible to blind the investigator or observer to the device being applied, this study is not a double-blind trial and the potential for bias may exist. Second, although all the intubations were performed by one person who had sufficient experience in use of GlideScope (more than 20 times), the investigator had vastly more experience with the Macintosh laryngoscope; this may affect the results as a confounding factor. Third, this study was conducted on normal patients and its results cannot be extrapolated to patients with hypertension, to those who are anticipated to have difficult orotracheal intubation or having other comorbidities. Finally, ideally invasive BP monitoring by inserting an arterial line could have been more informative to capture more frequent BP readings; however, it was unjustifiable to use invasive BP readings in our patient population for study purposes only.

In conclusion, in patients that were presumed to have normal airways, the GVL did not show any special advantages over the MDL in terms of the attenuation of hemodynamic changes following orotracheal intubation. The intubation time in the GlideScope group was significantly longer as compared to the Macintosh group suggesting that the advantages of GVL in terms of hemodynamic responses could be possibly revealed if the time taken for orotracheal intubation could be decreased in GlideScope group.
